# Bifurcation and Entropy Analysis of a Chaotic Spike Oscillator Circuit Based on the S-Switch

**DOI:** 10.3390/e24111693

**Published:** 2022-11-19

**Authors:** Petr Boriskov, Andrei Velichko, Nikolay Shilovsky, Maksim Belyaev

**Affiliations:** Institute of Physics and Technology, Petrozavodsk State University, 185910 Petrozavodsk, Russia

**Keywords:** chaotic oscillator, LIF neuron, entropy, pulse position modulation, reservoir computing

## Abstract

This paper presents a model and experimental study of a chaotic spike oscillator based on a leaky integrate-and-fire (LIF) neuron, which has a switching element with an S-type current-voltage characteristic (S-switch). The oscillator generates spikes of the S-switch in the form of chaotic pulse position modulation driven by the feedback with rate coding instability of LIF neuron. The oscillator model with piecewise function of the S-switch has resistive feedback using a second order filter. The oscillator circuit is built on four operational amplifiers and two field-effect transistors (MOSFETs) that form an S-switch based on a Schmitt trigger, an active RC filter and a matching amplifier. We investigate the bifurcation diagrams of the model and the circuit and calculate the entropy of oscillations. For the analog circuit, the “regular oscillation-chaos” transition is analysed in a series of tests initiated by a step voltage in the matching amplifier. Entropy values are used to estimate the average time for the transition of oscillations to chaos and the degree of signal correlation of the transition mode of different tests. Study results can be applied in various reservoir computing applications, for example, in choosing and configuring the LogNNet network reservoir circuits.

## 1. Introduction

Chaotic dynamics is widely represented in various fields of science and technology. In engineering, chaotic systems can be used in communication security systems [1,2,3] automatic control methods [4], device failure diagnostics [5], time series forecasting [6], and pattern recognition [7]. Circuits of chaotic signal generators are modelled in physics, biology, chemistry, and economics. In neuroscience, electronic circuits imitate real neurons based on various biosimilar models with chaotic dynamics [8,9,10].

A leaky integrate-and-fire (LIF) neuron [10,11,12,13,14,15] represents the simplest spiking neuron model, which is based on a threshold (switching) element and an RC integrator, and has multiple implementation methods, including methods based on CMOS technology [14,15]. A two-terminal element with an S-shaped I–V characteristic (S-IVC) [13] can be used as a switching element and is widely represented in electronics, for example, in silicon trigger diodes and thin-film structures based on oxides of transition metals (V, Nb, Ti and others) [16]. An S-switch with non-volatile memory, as in the Pt/TiO_2_/Pt structure [17], is classified as memristor, and has high potential for applications in neurotechnologies as an electronic component of neural circuits, including LIF models [18,19]. An S-switch with volatile memory can be built of various combinations of transistors [20], for example, as a complementary pair, where the base of one transistor is connected to the collector of another transistor. Using a Schmitt trigger and MOSFET, we developed an active S-switch with high switching stability (see, Section 2.2).

Reservoir Computing (RC) is nonlinear dynamics based neurotechnology, where chaotic physical systems can be directly involved in the computational process. Such systems can be of mechanical, optical, or electronic nature [21]. Echo state [22] and liquid state machines [23] are RC concepts that use reservoirs consisting of a large number of unstable dynamic subsystems (neurons) with random connections. Simplified RC architectures, where the reservoir consists of only one node, can be represented as a one-dimensional machine that has a set of virtual nodes with time-multiplexed states [24,25,26]. The LogNNet network is another concept of RC that uses discrete chaotic mapping and matrix transformation of input data [27,28]. The reservoir of this network transforms the input data vectors from one dimension to another in a unique way for a given chaotic mapping and can be configured with mapping parameters.

Good prediction and recognition results are shown by RCs where the reservoir dynamics is on the edge of chaos with a sharp transition from a regular regime to a chaotic one [21,22,23,24]. The transitions can be identified by the change from regular low-entropy oscillations to high-entropy oscillations of irregular (chaotic) dynamics. A preliminary entropy analysis of chaotic models can reveal the characteristics of their bifurcation behaviour and find the options for their optimal tuning in reservoir networks.

The use of physical oscillators in RC is problematic; however, this problem does not exist in their mathematical (completely computational) representation. The problem is the generation of well-repeatable (at least at the initial stage) excited states of the reservoir under the same action, or the same input signals of the network. Otherwise, the reservoir will not be able to transform the input signals to the network’s output layer preserving signals’ characteristics. In other words, the reservoir will not have dynamic memory. For example, the LogNNet network uses a repeating chaotic time series for each input data vector, and the repetitions are easily implemented using mathematical mappings. However, for physical chaotic oscillators, the repeatability of time series from run to run can be a problem that requires additional study.

In the current study, we present a new chaotic spike (LIF) oscillator and a bifurcation and entropy analysis of its dynamics. In entropy analysis, the entropy NNetEn based on the LogNNet network [29] is calculated. The advantage of this method is the high sensitivity of the entropy estimate to the irregularity of short time series. We investigate the stationary mode of oscillations of the oscillator’s mathematical model and the experimental (analogue) circuit based on this model. In the experimental circuit, we analyse the “regular oscillations-chaos” transient mode in a series of multiple tests. The study results can be used in RC applications, and in particular, when choosing and configuring the LogNNet network reservoir circuit.

## 2. Methods

### 2.1. Chaotic LIF Oscillator Model

**Figure 1:**
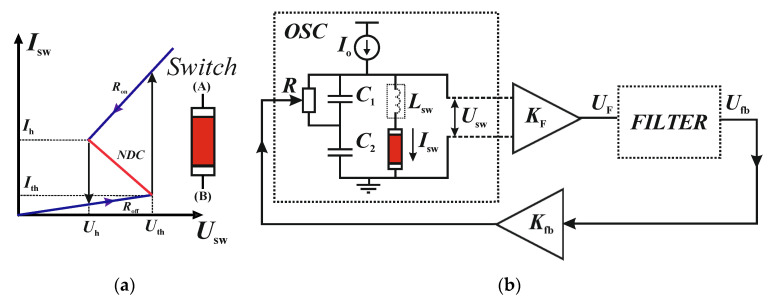
(**a**) S-IVC with NDC segments (red line). (**b**) General circuit of a chaotic LIF oscillator with feedback and two amplified modulus *K*_F_ and *K*_fb_ (*U*_F_ and *U*_fb_ are input and output voltages of *FILTER*). *U*_F_ can be proportional to both current *I*_sw_ and voltage *U*_sw_ of the switch. *L*_sw_ is noted by the dash as inner inductive element of the *OSC* block.

S-IVC has the following (piecewise linear) model of the dependence of current on voltage *I*_sw_(*U*_sw_) and the switching of states [13,30]:

(1) $$I_{\mathrm{sw}}(U_{\mathrm{sw}})\approx \left\{ \begin{array}{l} \frac{U_{\mathrm{sw}}}{R_{\mathrm{off}}},\;\mathrm{if}\;\mathrm{state}=\mathrm{OFF}\\ \frac{U_{\mathrm{sw}}}{R_{\mathrm{on}}},\;\mathrm{if}\;\mathrm{state}=\mathrm{ON} \end{array}\right.\;\mathrm{switching}\Rightarrow \left\{ \begin{array}{l} \mathrm{OFF}\to \mathrm{ON},\;\mathrm{if}\;U_{\mathrm{sw}}>U_{\mathrm{th}}\\ \mathrm{ON}\to \mathrm{OFF},\;\mathrm{if}\;U_{\mathrm{sw}}<U_{h} \end{array}\right.$$

where *R*_on_ and *R*_off_ are resistances of low (ON state) and high (OFF state) branches and *U*_th_ and *U*_h_ are threshold and holder voltages of the S-switch (Figure 1a).

When constructing a mathematical model of the LIF oscillator based on the S-switch, there is a problem associated with the ambiguity of the S-IVC function (1). This ambiguity can be eliminated by a small inductance *L*_sw_ connected in series to the switch (Figure 1b). Such inductance increases the dimension of the model, but makes it possible to use the inverse to (1), piecewise linear and single-valued function *U*_sw_(*I*_sw_) [13]):

(2) $$U_{\mathrm{sw}}(I_{\mathrm{sw}})=\frac{R_{\mathrm{on}}+R_{\mathrm{off}}}{2}I_{\mathrm{sw}}+\frac{R_{\mathrm{ndr}}-R_{\mathrm{off}}}{2}\left(\left|I_{\mathrm{sw}}-I_{\mathrm{th}}\right|-I_{\mathrm{th}}\right)-\frac{R_{\mathrm{ndr}}-R_{\mathrm{on}}}{2}\left(\left|I_{\mathrm{sw}}-I_{h}\right|-I_{h}\right)$$

with the resistance *R*_ndr_ in the negative differential conductance (NDC) section (Figure 1a):

(3) $$R_{\mathrm{ndr}}=\frac{U_{h}-U_{\mathrm{th}}}{I_{h}-I_{\mathrm{th}}}$$

where *I*_th_ = *U*_th_/*R*_off_ and *I*_h_ = *U*_h_/*R*_on_ are threshold and holder currents of the S-switch. The physical meaning of adding the inductance is the accounting for the inertia of the S-switch, that is, the finite time of the current change during the transitions between OFF and ON states.

Figure 1b presents the general concept of a chaotic LIF oscillator with an S-switch [30]. A basic spike oscillator (or LIF neuron), shown as an *OSC* block in Figure 1b, consists of two series capacitors *C*_1_ and *C*_2_ parallel to the switching element, and one of the capacitors (*C*_1_) has a parallel branch with a variable resistance *R*. The power supply of the circuit *I*_o_ sets the oscillator in self-oscillation mode: if the *I*_o_ value is in the NDC range, the LIF neuron generates regular impulses (spikes). A feature of the LIF neuron-oscillator is the dependence of spike frequencies *F* on the variable resistance *R* as a sigmoid type nonlinearity. The *F*(*R*) function has an inflection point at *R* = *R*_o_, where its second derivative is equal to zero [31].

The chaotic LIF oscillator (Figure 1b) has a feedback loop between the output signal of LIF neuron (current or voltage pulses of the S-switch) and variable resistance *R* through a second-order filter (*FILTER*, Figure 1b). In addition, two amplifying modules (with coefficients *K*_F_ and *K*_fb_) are connected in series with the filter (*U*_F_ and *U*_fb_ are input and output voltages of *FILTER*). These modules have the large input resistances, thereby eliminating the reverse effect of the filter on the LIF neuron (and vice versa). As modelled in [30], if the initial (reference) resistance value *R*(*t* = 0) = R* is set close to *R*_o_, and the parameters *K*_F_ and *K*_f_ (taking into account their sign) are chosen, the circuit of Figure 1b generates chaotic PPM oscillations.

Let us consider a variant, when the second-order filter of the circuit Figure 1b is a series oscillating LC circuit with conductance *C*_os_, inductance *L*_os_ and resistor *R*_os_. The input voltage of the filter (*U*_F_) is proportional with the coefficient *K*_F_ to the current of the switch (*U*_F_ = *K*_F_·*I*_sw_), and its output voltage (*U*_fb_) is the voltage on the capacitor *C*_os_.

Taking into account the infinitely large input resistances of the amplifying modules, based on the Kirchhoff laws, we obtain the following system of five differential equations for the circuit of Figure 1b:

(4) $$\left\{ \begin{array}{l} C_{1}\frac{{dU}_{1}}{dt}=I_{o}-I_{\mathrm{sw}}-S\left(U_{\mathrm{fb}}\right)U_{1}{;\;C}_{p}\frac{{dU}_{\mathrm{sum}}}{dt}=I_{o}-I_{\mathrm{sw}}-S\left(U_{\mathrm{fb}}\right)U_{1};\\ L_{\mathrm{sw}}\frac{{dI}_{\mathrm{sw}}}{dt}=U_{\mathrm{sum}}-U_{\mathrm{sw}}\left(I_{\mathrm{sw}}\right){;\;C}_{\mathrm{os}}\frac{{dU}_{\mathrm{fb}}}{dt}=I_{\mathrm{os}}{;\;L}_{\mathrm{os}}\frac{{dI}_{\mathrm{os}}}{dt}=K_{F}I_{\mathrm{sw}}-U_{\mathrm{fb}}-R_{\mathrm{os}}I_{\mathrm{os}} \end{array}\right.$$

where *U*_sum_ = *U*_1_ + *U*_2_ is the total voltage across capacitors *C*_1_ and *C*_2_, *C*_p_ = (*C*_1_+*C*_2_)/*C*_2_, *I*_os_ is the current in the LC circuit. In system (4), in addition to the non-linear (piecewise-linear) function of the switch *U*_sw_(*I*_sw_) (2), there is an inverse-linear function of the controlled resistance on the voltage *U*_fb_:

(5) $$S(U_{\mathrm{fb}})=\frac{1}{R_{\mathrm{rate}}+K_{\mathrm{fb}}U_{\mathrm{fb}}}$$

with the operating point *R*_rate_, which should be set to a value close to *R*_o_. The Table 1 lists all parameters of the S-switch and the circuit (Figure 1) that were used in [30] (expect for *L*_sw_) and in this study. For the parameters given in Table 1, the reference resistance is *R*_rate_~*R*_o_ = 190 Ω.

The limit *L*_sw_ → 0 corresponds to the condition that the current change during ON ⟷ OFF switch transitions is much shorter than the times of charging and discharging the capacitors *C*_1_ and *C*_2_ of the oscillator. In this case, in system (4), the third equation becomes a piecewise linear equation: *U*_sw_(*I*_sw_) = *U*_s_. In [30], model (4) is presented in a dimensionless form with time *t*′ = *t*/a_1_ = *t*/(*R*_os_·*C*_os_).

### 2.2. Chaotic LIF Oscillator Circuit

**Figure 2:**
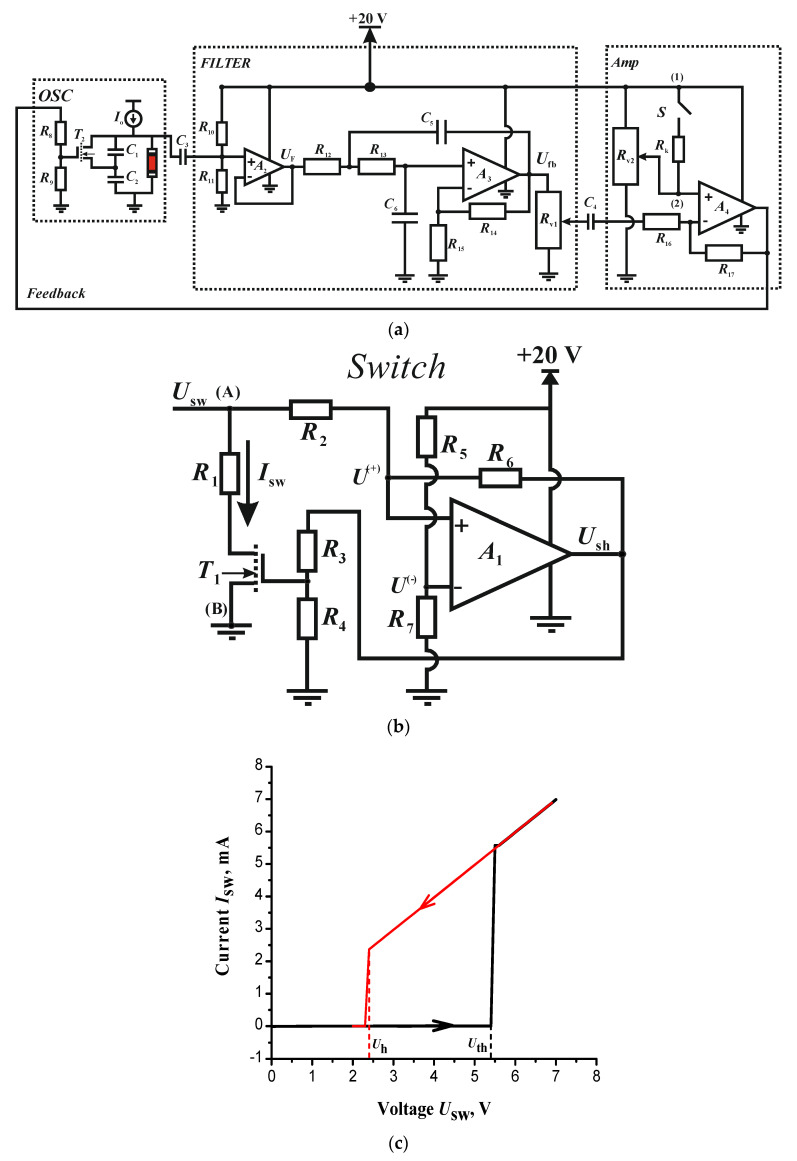
(**a**) Experimental chaotic LIF oscillator circuit based on operational amplifiers and MOSFETs. The circuit has additional branch with a switch *S* and resistance *R*_k_ between nodes (1) and (2) at the non-inverting input of the matching amplifier (*Amp*). The circuit (**b**) and experimental IVC (**c**) of the S-switch based on Schmitt trigger and MOSFET *T*_1_. (A) and (B) in figure (**b**) are terminals, similar to Figure 1a. The black and red lines in figure (**c**) are the ascending and descending of IVC branches.

The non-inductive analog circuit (Figure 2a) is based on the model circuit of Figure 1b and consists of three blocks: *OSC*, *FILTER* and *Amp* connected in feedback loop. The block *OSC* has a supply current *I*_o_. The blocks *FILTER* and *Amp* have 20 V supply voltages. All parameters of the circuit (Figure 2) used in the current study are listed in Table 2.

The block *OSC* is a basic spike neuron-oscillator, similar as in Figure 1b, but without the inductance (*L*_sw_) in the switch circuit. The switch is formed as an active two-terminal circuit based on the Schmitt trigger (comparator *A*_1_) in combination with the MOSFET *T*_1_ (Figure 2b). The output signal of the trigger (*U*_sh_(*t*)) are rectangular pulses, suitable for digital processing.

The S-shaped IVC of the circuit Figure 2b is formed as follows. The voltage at the non-inverting input (*U*^(+)^) of comparator *A*_1_ changes over time in two ways:

(6) $$U^{\left(+\right)}(t)=\left\{ \begin{array}{l} U_{1}^{\left(+\right)}(t)=\frac{U_{\mathrm{sw}}(t)\cdot R_{6}+U^{\left(1\right)}\cdot R_{2}}{R_{2}+R_{6}},\;\mathrm{if}{\;U}_{\mathrm{sh}}=U^{\left(1\right)}\\ U_{2}^{\left(+\right)}(t)=\frac{U_{\mathrm{sw}}(t)\cdot R_{6}+U^{\left(2\right)}\cdot R_{2}}{R_{2}+R_{6}},\;\mathrm{if}{\;U}_{\mathrm{sh}}=U^{\left(2\right)} \end{array}\right.$$

since the voltage *U*_sh_ can take only two values: low level *U*^(1)^ and high level *U*^(2)^. If *U*_sh_ = *U*^(1)^ and the voltage *U*^(+)^(*t*) = *U*_1_^(+)^(*t*) reaches the value *U*_ref_, which is constant voltage of inverting input *U*^(−)^ (Figure 2b), then *U*_sh_ sharply changes from *U*^(1)^ to *U*^(2)^. The voltage at the gate of the transistor *T*_1_ changes through the divider *R*_3_–*R*_4_ and *T*_1_ opens. Then, the current *I_s_*_w_ of the switch (through the resistance *R*_1_) will cause a surge of the transition OFF to ON state, the capacitances *C*_1_ and *C*_2_ of the oscillator are discharged through the open transistor and the voltage *U*_sw_(*t*) drops. When the voltage *U*^(+)^(*t*) = *U*_2_^(+)^(*t*) reaches the value *U*_ref_, the output voltage of the trigger *U*_sh_ again switches to the value *U*^(1)^ and the transistor closes. From this moment, *C*_1_ and *C*_2_ are charged by the supply current *I*_o_, the voltage *U*_sw_(*t*) increases, and the trigger switching process is repeated. Figure 2c demonstrates the experimental S-IVC of the switch based on Schmitt trigger using circuit parameters from Table 2, which give the following switching parameters: *U*_th_~5.4 V, *U*_h_~2.4 V, *R*_on_~1 kΩ and *R*_off_~600 kΩ.

The second block (*FILTER*, Figure 2a) consists of the voltage follower *A*_2_ and active second-order RC filter (Sullen-Kay filter) based on the amplifier *A*_3_ [32]. The follower *A*_2_ is the first module of the model circuit (Figure 2a) with *K*_F_ = 1, and it excludes the reverse effect of the filter on the LIF neuron. The third block (*Amp*) of Figure 2a is a matching inverting amplifier *A*_4_. It corresponds to the second module of the model circuit with *K*_fb_ = −*R*_17_/*R*_16_ and is connected to the LIF neuron through a divider (*R*_8_–*R*_9_) at the gate of the MOSFET *T*_2_. In addition to the model circuit (Figure 1b), there are isolation capacitors *C*_3_ and *C*_4_ in the circuit of Figure 2a to cut off the constant components (DC voltage) of the signals between the blocks.

The circuit on Figure 2a has two control resistances: *R*_v1_ and *R*_v2_. The first resistance *R*_v1_ changes the amplitude of the output voltage *U*_fb_(*t*) of *FILTER* block, the RMS value *U*_fbo_ of which varies from 0 to 10 V, or up to half the supply voltage, as *R*_14_ = *R*_15_ (see Table 2)). The second resistance *R*_v2_ on the non-inverting input of *A*_4_ (*Amp*) modifies the DC component of the feedback signal and sets the MOSFET reference voltage *T*_2_.

The transition between different oscillation modes in the circuit Figure 2c can be triggered by changing the DC voltage at the non-inverting input of the matching amplifier *A*_4_, adjusting the resistance *R*_v2_. For a sharp transition between two oscillation modes, we add an additional branch (between nodes (1) and (2) Figure 2a) with resistance *R*_k_ and electronic switch *S*. In the transient test series (see Section 3.2.2), the circuit was set to regular mode when switch *S* is open. After the circuit reaches stationary periodic oscillations, closing the switch *S* initiates a stepped voltage pulse and the transition of the oscillations to the chaos mode.

### 2.3. LogNNet and Entropy NNetEN of Chaotic Time Series

Although LogNNet is a reservoir neural network, it differs from traditional RC in a way the reservoir is formed. In traditional RC, the reservoir consists of a set of neurons with sigmoid (echo state [22]) or threshold (liquid state machines [23]) activation functions and connected by random connections. The matrix of connections between neurons and the input layer is fixed, and the output signals of the reservoir are read from a certain set of neurons (nodes). In LogNet [27,28], the hidden layer (reservoir) has a convolution (matrix) transformation to transfer input data vectors to the output layer (classifier) with a change in the dimension of their representation space. The kernel of the transformation is formed by setting the reservoir matrix through generating a discrete chaotic mapping with certain initial data and parameters. As in traditional RC, only the weight matrix of the output layer is trained in LogNNet, and the parameters of the reservoir are adjusted through the selection of chaotic display parameters.

LogNNet is applied to calculate NNetEn entropy of time series [29]. The LogNNet model [27] was originally designed for recognizing handwritten digits (28 × 28 = 784 pixels) in the MNIST dataset [33]. Optimizing LogNNet for this task, it was found [30] that the accuracy of digit recognition directly correlates with the values of the approximate entropy (ApEN) [29] of the time series that is generated by the chaotic discrete reservoir mapping. By tuning the network, it is possible to achieve a high degree of coincidence of the recognition accuracy and ApEN values in a certain parameter range. The method for estimating the entropy of an arbitrary chaotic sequence using LogNNet is to set a reservoir matrix based on this sequence and calculate the accuracy of recognition of handwritten letters from the MNIST dataset. Therefore, classification accuracy (0 ÷ 100%) is considered to be the entropy measure NNetEn (0÷1) as *classification accuracy*/100%.

**Figure 3:**
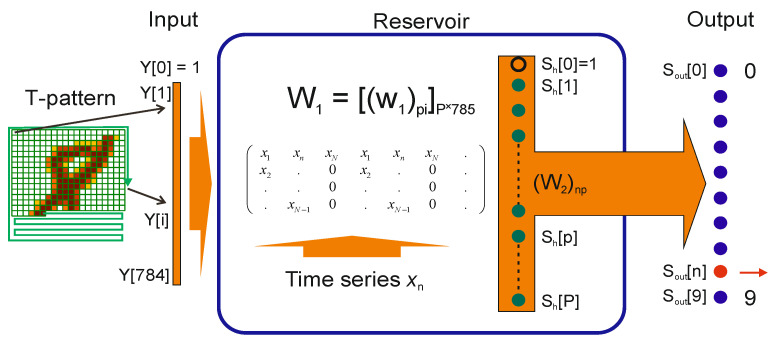
The LogNNet structure [27].

The main advantage of this method for estimating the entropy of signals is the simplicity and high sensitivity to the irregularity of short time series. Figure 3 reflects the LogNNeT concept of NNetEN entropy calculation for time series *x*_n_, which has the following algorithm: (1) Loading the MNIST-10 database; (2) loading time series *x*_n_; (3) the reservoir matrix *W*_1_ is constructed using the given time series (Method 3 [34]); (4) the training process of the LogNNet 784:25:10 network is performed on a training set; (5) the testing process of the LogNNet 784:25:10 network is performed on a test set, classification accuracy and entropy NNetEn are calculated.

In this algorithm, a data set from MNIST 10 is fed to the LogNNet (Input) as a greyscaled and normalised input vector Y = {Y [0], Y [1], …, Y [784]}. The reservoir uses the matrix W_1_ to transform the input vectors Y into output vectors S_h_, which contains *N* × *P* = 19,625 elements, where *N* = 785 is the number of components of input vectors Y and P = 25 is the number of components of output vectors S_h_. The vectors S_h_ with the connection matrix W_2_ are fed to the output layer (Output), consisting of 10 neurons S_out_ (digits 0 ÷ 9). Training the network to recognize digits from the MNIST 10 database (with 100 training epochs), a linear classifier is used to tune only the weight matrix W_2_. To calculate the non-stationary signal entropy, for example, its transient response, a data window is extracted from the time series with the number of elements *N* = 100. As the window slides over the time series, NNetEn values are calculated to find the dependence of entropy on time.

## 3. Results

### 3.1. Chaotic Oscillator Model

**Figure 4:**
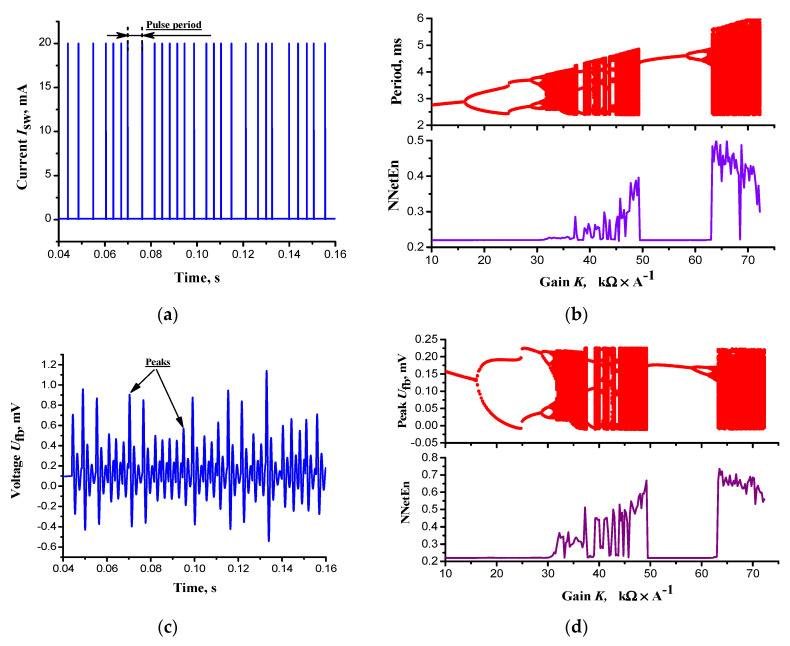
Oscillograms and bifurcation diagrams of the model Figure 1b: (**a**,**b**) switch current pulses *I*_sw_(*t*), pulses’ periods and entropy (NNetEnt); (**c**,**d**) LC filter output voltage *U*_fb_(*t*), voltage’s peaks and entropy (NNetEnt). Calculation parameters are listed in Table 1, expect for *K*_fb_ in (**b**,**d**): *K*_fb_ = –10 ÷ 75 kΩ·V^−1^.

The simulation of model circuit on Figure 1b, performed by the MatLab software (method ode23s, see the program in Supplementary Material) based on Equation (4), demonstrates the existence of a chaotic mode of PPM for switch current pulses *I*_sw_(*t*) and the chaotic oscillation of output voltage of the filter *U*_fb_(*t*). Figure 4 presents oscillograms, bifurcation diagrams and entropy calculation, where the coefficient *K* = |*K*_F_·*K*_fb_| is the variable parameter. The model circuit dynamics depends on the product of the coefficients *K*_F_ and *K*_fb_ of the amplifying modules, as the modules are connected in series and have infinitely large input resistances. In addition, modules can invert the signal, i.e., *K*_F_ and(or) *K*_fb_ can be less than zero.

Bifurcation diagrams for periods between the switch current pulses (Figure 4a) show that the transition to the dynamic chaos of PPM occurs according to the scenario of a period-doubling bifurcation cascades. Chaos periods (completely filled periods of values) alternate with periods where a finite number of pulses are observed, that is, regular dynamics. The bifurcation diagram for output voltage of the filter *U*_fb_(*t*) (Figure 4d), recalculated on its peaks (Figure 4c), demonstrates the similar dynamics. The entropy value (NNetEn) for both signals increases sharply upon transition to the dynamic chaos mode, and its changes repeat the alternation of chaos periods (large entropy values) and regular dynamics (small entropy values).

### 3.2. Chaotic Oscillator Circuit

#### 3.2.1. Steady State Analysis

**Figure 5:**
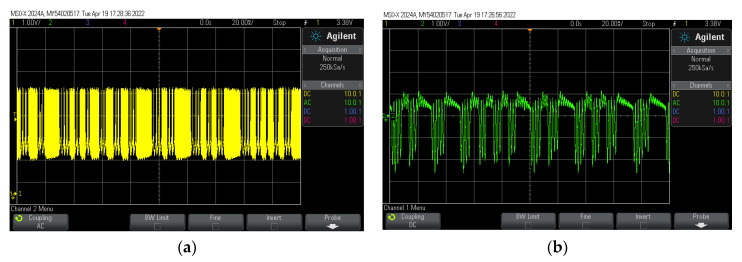
Photos of voltage oscillograms: (**a**) voltage *U*_sw_(*t*) of the S-switch; (**b**) output voltage *U*_fb_(*t*) of the Sullen-Kay filter. Circuit parameters are listed in Table 2.

Photos of voltage oscillograms in the stationary (steady) chaotic mode of the switch (*U*_sw_(*t*)) and filter (*U*_fb_(*t*)) of the circuit Figure 2 are presented in Figure 5. The sequences of switch pulses (*U*_sw_(*t*), Figure 5a) have a chaotic burst form, in other words, they represent packets (bursts) of rapidly oscillating spikes, randomly changing in duration and intervals of low-frequency activity. This chaotic nature of the oscillations of the experimental circuit is the result of its tuning, when the active filter is in a strong excitation mode with large oscillation amplitudes, and the MOSFET *T*_2_ (Figure 2a) often switches between fully open and closed states. The oscillations of *U*_fb_(*t*) with small amplitudes are replaced by oscillations with large amplitudes (Figure 5b).

**Figure 6:**
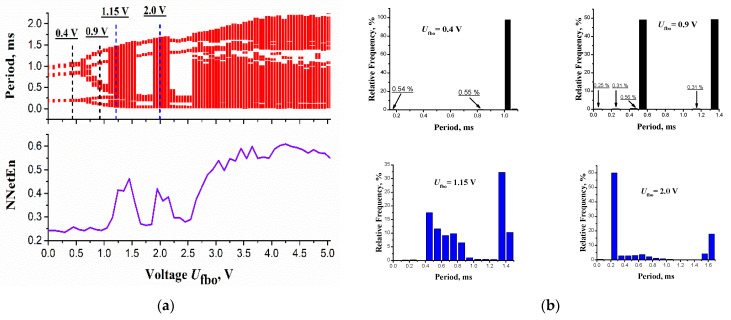
(**a**) Bifurcation diagram of the pulse periods of the circuit (Figure 2a) and dependence of pulses’ entropy NNetEn on the RMS (root mean square) output voltage of the Sallen-Key filter *U*_fbo_; (**b**) period distribution histograms for four *U*_fbo_ values.

Bifurcation diagrams of the switch pulses’ periods and entropy (NNetEn) with a change in the RMS output voltage of the filter (*U*_fbo_) are shown in Figure 6a. The values *U*_fbo_ were changed by the variable resistance *R*_v1_ (Figure 2a). The diagram has windows with a finite number of pulses, which alternate with windows of completely filled periods. On the initial small range of values *U*_fbo_, the switch pulses have three periods, but only one period has frequently repeated signal values. This is demonstrated by the histogram of the relative frequency distribution of periods with the value *U*_fbo_ = 0.4 V (Figure 6b, upper left figure), where one period has a frequency probability of ~99%. As *U*_fbo_ increases, regular single-mode pulse sequences transform into two-mode sequences. For *U*_fbo_ = 0.9 V (Figure 6b, upper right figure), out of six periods, two periods have the frequency probability ~50%, and the remaining four periods have the frequency probability less than 1%. As in the model (Figure 4b), we witness the period doubling scenario in the experimental circuit of the LIF oscillator. However, there are periods of signals in the circuit, which are the result of rare switching not predicted by the model. We suppose this switching is the result of the noise of the circuit elements, primarily MOSFETs *T*_1_ and *T*_2_.

On the bifurcation diagram, where there is a continuous filling of the periods’ values (the lower histograms on Figure 6b with *U*_fbo_ = 1.15 and 2.0 V), the distribution of distances between pulses is highly nonuniform with a dip in the frequency probability between the two dominant periods. This irregularity of PPM on the experimental setup is explained by the burst nature of the oscillations (see Figure 5), when the spectrum is dominated by two frequency components that are very different from each other. The entropy NNetEn dependence on the values of *U*_fbo_ (Figure 6a, bottom) is similar to the alternation of regularity and chaos of the bifurcation diagram of the circuit, however, its shape is smoother than the dependence of the model (Figure 4b). This is a consequence of the limited data on the pulse periods obtained in the experimental circuit in comparison with the model calculation. In addition, noise in the circuit leads to period fluctuations and blurring of the fine bifurcation structure of entropy.

#### 3.2.2. Transient State Analysis

**Figure 7:**
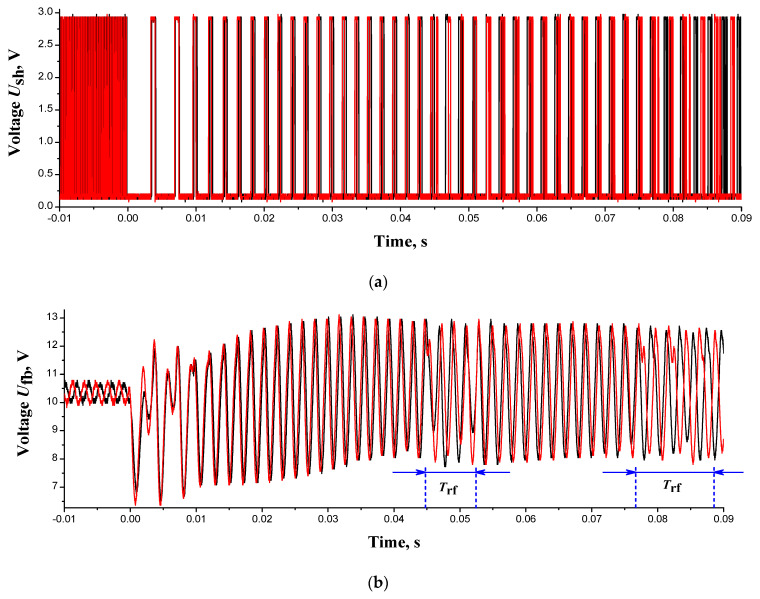
Experimental voltage oscillograms of transient mode: (**a**) voltage *U*_sh_*(t*) of Schmitt trigger of the S-switch (Figure 2b); (**b**) output voltage *U*_fb_(*t*) of the Sullen-Kay filter. The red and black oscillograms correspond to two different tests. Figure (**b**) shows the regularity failure intervals (*T*_rf_).

Transient mode “regular oscillations–chaos” of the experimental circuit, Figure 2a, was generated by closing the switch S in a series of 10 tests. Figure 7a,b reflect the pulse oscillograms of two tests of the Schmitt trigger *U*_sh_(*t*) and the Sallen-Key filter output voltage *U*_fb_(*t*), respectively. Oscillograms demonstrate the time (*t* = 0 s) of the switch closing and the beginning of the “regular oscillations–chaos” transition in the time range *t* = 0 ÷ 0.09 s. Before the transition, at *t* < 0, the regular oscillations *U*_fb_(*t*) of the test oscillograms have a constant phase shift. It is explained by the fact that the switch closure for each test occurred at a random time and the oscillograms are independent of each other.

From the moment of transition (*t* = 0), the phase shift between any oscillograms *U*_fb_(*t*) from the test series almost disappears, and the oscillograms become similar to periodic oscillations with regularity failures in intervals *T*_rf_, as shown in Figure 7b. These failures of regularity increase with time, and later, the quasi-regular dynamics of the circuit disappears completely: the oscillations *U*_fb_(*t*) and pulse periods *U*_sh_(*t*) reach the chaotic mode.

**Figure 8:**
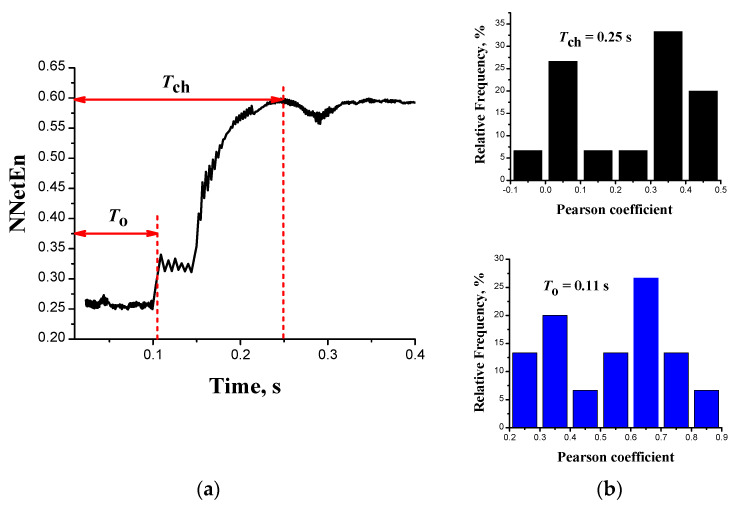
(**a**) Transient characteristic of average entropy (NNetEn) for pulse periods *U*_sh_*(t*) in a series of 10 tests; (**b**) Pearson correlation distributions for signals *U*_fb_(*t*) in time intervals [0, *T*_ch_] (upper histogram) and [0, *T*_o_] (lower histogram).

Entropy NNetEn of oscillograms estimates the settling time of the chaotic dynamics *T*_ch_ as the time from the beginning of the transition (closing the switch) to the time of the maximum signal entropy level. Figure 8a shows the transient response of the average signal entropy *U*_sh_(*t*) over 10 tests. A small oscillating plateau on the NNetEn curve starts from the time *T*_o_~0.11 s. Before that time, the entropy has a low value around 0.25. From the time *t* > 0.15 s, the entropy begins to increase sharply, and by the time *t* = *T*_ch_, the entropy reaches its maximum level (~0.6), corresponding to stationary chaotic dynamics.

The entropy NNetEn of signals estimates the settling time of the chaotic dynamics Tch in the circuit Figure 2a as the time from the beginning of the transition (closing the switch) to the time of the maximum level of signal entropy. Figure 8a demonstrates the average entropy over 10 tests of pulse period *U*_sh_(*t*). A small oscillating plateau on the NNetEn curve starts from the time *T*_o_~0.11 s, and before that time, the entropy has a low value~0.25. From the time *t* > 0.15 s, the entropy increases sharply, and by the time *t* = *T*_ch_, the entropy reaches its maximum level (~0.6), corresponding to the maximum chaotic dynamics.

For a quantitative analysis of the repeatability of signals from different tests, we calculate the paired Pearson correlation coefficients (*Pear*) for the values of the oscillograms *U*_fb_(*t*). Figure 8b demonstrates the distributions of the coefficients *Pear* for 10 trials (45 combinations in total) from the beginning of the transition (*t* = 0) to the times *t* = *T*_o_ and *t* = *T*_ch_. Closer to the chaotic regime (*t* = *T*_ch_), the correlation between the test signals decreases significantly. The Pearson coefficient does not exceed 0.5 and has a significant proportion (>30%) of signal pairs, where *Pear* is less than 0.1. On the contrary, for the initial transition interval [0, *T*_o_], all coefficients *Pear* in the series exceed 0.2, and a significant proportion (>45%) of signal pairs have a correlation value above 0.6.

The low correlation between the signals of different tests by the moment of transition to the chaotic mode (*t* = *T*_ch_) indicates the accumulation of large fluctuations in the circuit. As a result, the phase trajectories of the oscillations are significantly moving away from each other. Such fluctuations can be stochastic switching of the oscillator, as a consequence of the internal noise of the circuit elements, especially MOSFETs *T*_1_ and *T*_2_. Thus, the signals generated by the chaotic LIF oscillator are not repeated from test to test at *t* > *T*_ch_, and this part of the oscillograms cannot be used in RC.

## 4. Discussion

Our study shows that the calculation of the entropy of oscillations effectively complements the traditional bifurcation analysis to identify the features of nonlinear dynamics and adjust the operating modes of chaotic circuits. For example, the chaos generator can be tuned to various parameter ranges, including the edge of chaos, where there is a sharp transition of entropy from a low level of the regular mode to high values corresponding to strong chaotic dynamics. In addition, entropy analysis can be a simple and effective tool for studying transient modes of nonlinear dynamics of chaotic circuits. This is demonstrated by the example of the transition “regular oscillations-chaos”, where, using the calculation of entropy, we estimate the average time for the transition to chaos and the degree of signal correlation of the transition mode of different tests.

The combination of bifurcation analysis with the study of transient modes of nonlinear models is applied to construct the continuation of bifurcation diagrams. In this case, the initial conditions are not fixed, but the analysis starts from the end point of the trajectory calculated using previous bifurcation parameters. By comparing several continuation diagrams, possible areas of model multistability are estimated, in other words, the coexistence of attractors with the same parameters but different initial conditions is investigated [35,36]. Similar simulation studies were conducted for chaotic generators based on memristors [37,38]. This analysis is highly relevant for the future research, as reservoir-computing applications require repeatability and the absence of multistability of chaotic signals.

The presented model circuit of the chaotic spike oscillator (Figure 1b) is a voltage-controlled relaxation oscillator (VCO), where the feedback signal passes through a second-order filter tuned to an oscillating (self-excited) mode. Unlike conventional VCOs, where the frequency of the signal is controlled linearly by the input voltage, in the LIF oscillator, the frequency control function (through a variable resistance) is not linear and has an inflection point with a zero second derivative.

In addition, this model is an example of a neuromorphic chaotic circuit of a simple spike LIF neuron. Previously, LIF neurons with chaotic dynamics were implemented either using the time-varying injected current combined with a periodic modulation of parameters [39] or using the quadratic resistance nonlinearity in the RC neuron integrator [10]. In our case, the nonlinearity is not created by external elements (with the exception of the S-switch), but nonlinearity is inherent in the internal properties of the oscillator, namely, in the high-speed coding of the output pulses.

Although the chaotic oscillator circuit is built on simple non-precise operational amplifiers and transistors, it demonstrates high stability of regular mode oscillations. However, the differences in the dynamics of an ideal model oscillator and its experimental implementation exists in the chaotic regime. This is caused by, first, the use of a MOSFET transistor in the experimental circuit Figure 2 as a controlled resistance. The MOSFET *T*_2_ has a narrow control range because it often has either fully open or closed state in the active filter mode due to strong fluctuations in the amplitudes of the output signal. These unstable (random) transistor switchings determine the chaotic oscillating dynamics of the circuit, which, in contrast to the dynamics of the model, is close to the burst type. Second, the internal noise of the circuit elements affect the chaotic dynamics. Compared to the model calculation, the bifurcation diagram is blurred (Figure 6a) and fluctuations in the circuit during “regular oscillations-chaos” transition are accumulated.

The neural network LogNNet uses a discrete chaotic mapping, which can be replaced by any chaotic circuit, for example, an analog oscillator presented in this study. In the LogNNet algorithm, a discrete mapping or time-sampled analog chaos generator generates a matrix of input weights that forms the kernel to transform the input space to the higher-dimensional feature space. First, this matrix can be set once and its copy can be used during the entire execution of the neural network task. A second option is to start generating the matrix for each input data vector. The first method requires the inclusion of memory cells in the LogNNet hardware. For portable devices, used as ambient intelligence in the Internet of Things environment, this can be a significant drawback, limiting the amount of data being processed. In the second case, errors may occur in the repetition of the generator chaotic signal from one start to another start. Nevertheless, in this study, we have demonstrated that it is possible to use the transient mode in a short time range *t* < *T*_o_, where a high repeatability of the chaotic signal from start to start is observed. The study results can be used to create a digital-to-analogue prototype of the LogNNet reservoir with the chaos generator based on the presented circuit.

## 5. Conclusions

The proposed circuit of the spike LIF generator has a chaotic dynamic of the pulse position modulation controlled by feedback with frequency coding of instability firing rate. The model and the experimental circuit are compared using the one-parameter bifurcation analysis together with the calculation of the entropy of oscillations. The new method based on entropy calculation is developed for estimating the transition time from the initial stationary regular regime to chaos for chaotic circuits. We hope the study results will encourage the future research of the proposed chaotic model in terms of circuit design and detailed mathematical analysis, as well as promote developing applications based on findings, including neuromorphic computing.

## Figures and Tables

**Table 1:** Parameters of the S-switch and model circuit (Figure 1).

S-Switch											
*U*_th_, *I*_th_	*U*_h_, *I*_h_	*R*_on_	*R*_off_	*I*_o_	*C*_1_, *C*_2_	*L*_sw_	*K*_F_	*K*_fb_	*L*_os_	*R*_os_	*C*_os_
4 V 0.1 mA	2 V 10 mA	200 Ω	40 kΩ	0.15 mA	0.01 µF 1 µF	0.1 µH	1 V·A^−1^	−100 kΩ·V^−1^	1 mH	1 Ω	100 µF

**Table 2:** Parameters of the analog circuit (Figure 2).

Schmitt Trigger S-Switch (Figure 2b)			*I*_o_	*C*_1_, *C*_2_	*C*_3_, *C*_4_, *C*_5_, *C*_6_	*R*_8_, *R*_9_	*R*_10_, *R*_11_	*R*_12_, *R*_13_	*R*_14_, *R*_15_	*R*_16_, *R*_17_	*R*_V1_, *R*_V2_	*R*_k_
*R*_1_, *R*_2_	*R*_3_, *R*_4_	*R*_5_, *R*_6_, *R*_7_										
1 kΩ 100 kΩ	39 kΩ	300 kΩ 500 kΩ 100 kΩ	0.5 mA	22 nF 1 µF	0.1 µF	1 kΩ 100 kΩ	510 kΩ 180 kΩ	20 kΩ 0.5 kΩ	10 kΩ	100 kΩ	0 ÷ 10 kΩ 0 ÷ 50 kΩ	10 kΩ
Amplifiers *A*_1_–*A*_4_						MOSFETs *T*_1_ and *T*_2_						
TL082CP						ZVN2120						

## Data Availability

The database of handwritten digits MNIST-10 (available on Yan LeCun’s Internet page [31]) was used for the study.

## Supplementary Materials

The following supporting information can be downloaded at: https://www.mdpi.com/article/10.3390/e24111693/s1, Program_LIF_osc.pdf: calculation program in the MathLab.
