# Supplementary material for: Bifurcation and Entropy Analysis of a Chaotic Spike Oscillator Circuit Based on the S-Switch
Source: Entropy (Basel). 2022 Nov 19;24(11):1693. doi: 10.3390/e24111693 (PMC9689857; doi:10.3390/e24111693)
Supplement: Supplementary file 1 [file entropy-24-01693-s001.zip › entropy-2028420-supplementary.pdf]

The system of equations (4) with S-switch (2) and variable resistor (5) functions is transformed to a dimensionless form:

$$\dot{x} = K_1\left(z - f_1(x)\right),\ \dot{y} = 1 - x - f_2(q)y,\ \dot{q} = K_3 w,$$
$$\dot{z} = K_2\left(1-x\right) - f_2(q)y,\ \ \dot{w} = K_4\left(K_5 x - q\right) - K_3 K_4 w, \tag{S1}$$

$$f_1(x) = 0.5\Big(alpha\cdot\ x + gamma1\cdot\ \left(\left|x - I_1\right| - I_1\right) - gamma2\cdot\ \left(\left|x - I_2\right| - I_2\right)\Big),$$
$$f_2(q) = \left(R_s + K_6 q\right)^{-1}. \tag{S2}$$

Dimensionless variables and parameters of the switch and system (S1, S2) are shown in Table S1.

Table S1. Dimensionless variables, parameters of the S-switch and the system (S1, S2). $a_1 = \tau$ is a time constant, t/τ is dimensionless time of (S1).

Variables	Parameters		
$x = I_{sw}/I_o$	$U_o = a_1 \cdot I_o/C_1$		$K_4 = a_1^2/(K_2 \cdot a_2)$
$y = U_1/U_o$	$K_1 = a_1^2/(L_{sw} \cdot C_1)$		$K_5 = K_F \cdot C_1/a_1$
$z = U_{sum}/U_o$	$K_2 = (C_2+C_1)/C_2$		$K_6 = K_{fb} \cdot I_o$
$q = U_{fb}/U_o$	$K_3 = C_1/C_{os}$		$R_s = R^* \cdot C_1/a_1$
$w = I_L/I_o$	S-switch		
LC Filter	$R_1 = R_{off} \cdot C_1/a_1$		$I_1 = I_{th}/I_o$
$a_1 = R_{os} \cdot C_{os}$	$R_2 = R_{on} \cdot C_1/a_1$		$I_2 = I_h/I_o$
$a_2 = L_{os} \cdot C_{os}$	$R_{ndr} = C_1/a_1 \cdot (U_{th} - U_h)/(I_{th} - I_h)$		
	$alpha = R_1 + R_2$	$gamma1 = R_{ndr} - R_1$	$gamma2 = R_{ndr} - R_2$

% System global parameters (S1, S2)

global *K*1 *K*2 *K*3 *K*4 *K*5 *K*6 *alpha gamma*1 *gamma*2 *R*s *I*1 *I*2

% Switch parameters (dimensional, SI units)

*U*h=2; *U*th=4; *I*th=1e-4; *I*h=1e-2; *R*on=200; *R*off=4e4;

% Parameters of system (4) and functions (2) and (5) (dimensional, SI units)

*I*o=1.5e-4; *L*sw=1e-9; *C*os=1e-4; *L*os=1e-3; *R*os=1; *R*o=190;

*C*1=5e-9; *C*2=1e-6; *a*1=Ros*Cos; *a*2=*L*os**C*os;

% Finite (dimensionless) simulation time. Real calculation time (s): *t=Tend***a*1

Tend =1000;

% Dimensionless parameters of the system of equations (S1, S2)

A=*a*1/*C*1; *U*o=*I*o**A*; *R*s=*R*o/*A*; *K*F=1; *K*fb=-10e4;

*K*1=*a*1**A*/*L*sw; *K*2=(*C*1+*C*2)/*C*2; *K*3=*C*1/*C*os; *K*4=*a*1^2/(*a*2**K*3); *K*5=*K*F/*A*; *K*6=*K*fb**I*o;

*R*ndr=(*U*h-*U*th)/((*I*h-*I*th)**A*); *R*1=*R*off/A; *R*2=*R*on/*A*; *I*1=*I*th/*I*o; *I2*=*I*h/*I*o;

alpha=*R*1+*R*2; *gamma*1=*R*ndr-*R*1; *gamma*2=*R*ndr-*R*2;

```
% Initial conditions and simulation parameters
y0 = [0;0;0;0;0];options = odeset( 'RelTol', 1e-3,'AbsTol', 1e-6, 'Maxstep', 0.001);
% The calculation of system (S1) by the ode23s method, where the module Swich_Res is used
[t, y] = ode23s('Swich_Res', [0 Tend], y0,options);

% The transition to dimensional variables
Time=t*a1; Isw=y(:,1)*Io; % The current of the switch
Ufb=y(:,4)*Uo; % The voltage of LC filter
% Filter voltage and switch current oscillograms
plot(Time, Ufb, 'r.-');grid on; plot(Time, Isw, 'b.-');grid on; figure;

% S-switch function (S2)
function  Sw = F1(x)
global alpha gamma1 gamma2 I1 I2
Sw=0.5*alpha*x+0.5*gamma1*(abs(x-I1)-I1)-0.5*gamma2*(abs(x-I2)-I2);
end;

% Variable resistor function (S2)
function  Res = F2(x)
global Rs K6
Res=1/(Rs+K6*x);
end;

% The module function for calculation of system (S1)
function F = Swich_Res(t, y)
global K1 K2 K3 K4 K5;
F =[K1*(y(3)-F1(y(1))); 1-y(1)- F2(y(4))*y(2);K2*(1-y(1)) - F2(y(4))*y(2);K3*y(5);K4*(K5*y(1)-y(4))-K3*K4*y(5)];
end;
```
